# Na^+^/H^+^ Exchangers Induce Autophagy in Neurons and Inhibit Polyglutamine-Induced Aggregate Formation

**DOI:** 10.1371/journal.pone.0081313

**Published:** 2013-11-21

**Authors:** Kazuya Togashi, Shuji Wakatsuki, Akiko Furuno, Shinji Tokunaga, Yoshitaka Nagai, Toshiyuki Araki

**Affiliations:** 1 Department of Peripheral Nervous System Research, National Institute of Neuroscience, National Center of Neurology and Psychiatry, Kodaira, Tokyo, Japan; 2 Department of Degenerative Neurological Diseases, National Institute of Neuroscience, National Center of Neurology and Psychiatry, Kodaira, Tokyo, Japan; National Cancer Center Research Institute, Japan

## Abstract

In polyglutamine diseases, an abnormally elongated polyglutamine results in protein misfolding and accumulation of intracellular aggregates. Autophagy is a major cellular degradative pathway responsible for eliminating unnecessary proteins, including polyglutamine aggregates. Basal autophagy constitutively occurs at low levels in cells for the performance of homeostatic function, but the regulatory mechanism for basal autophagy remains elusive. Here we show that the Na^+^/H^+^ exchanger (NHE) family of ion transporters affect autophagy in a neuron-like cell line (Neuro-2a cells). We showed that expression of NHE1 and NHE5 is correlated to polyglutamine accumulation levels in a cellular model of Huntington's disease, a fatal neurodegenerative disorder characterized by accumulation of polyglutamine-containing aggregate formation in the brain. Furthermore, we showed that loss of NHE5 results in increased polyglutamine accumulation in an animal model of Huntington's disease. Our data suggest that cellular pH regulation by NHE1 and NHE5 plays a role in regulating basal autophagy and thereby promotes autophagy-mediated degradation of proteins including polyglutamine aggregates.

## Introduction

Accumulation and aggregation of mutant proteins is a characteristic feature of a number of neurodegenerative disorders, including Parkinson's disease and Huntington's disease (HD) [1]. In HD, for example, the disease-causing mutation in huntingtin (HTT), a protein of uncertain function causes expansion of a stretch of glutamines (polyQ) near its N terminus, and the mutant form of HTT accumulates as nuclear and cytoplasmic inclusions in an HD brain [2]. One of the major therapeutic challenges in the field of neurodegeneration has been to improve the degradation of accumulated mutant proteins.

Autophagy is a cellular protein clearance mechanism and can, in principle, clear aggregation-prone proteins [3]. In this process, double-membrane organelles, called autophagosomes, engulf cellular proteins and organelles and fuse with lysosomes to form autolysosomes, which then degrade the organelle's contents [3]. Some previous reports showed that mutated HTT expression may be associated with up-regulated autophagy, and autophagy degrades polyQ-expanded proteins [4], [5].

Induction of autophagy is typically observed by nutrient deprivation in many types of cells and organs and promote protein turnover to combat against starvation [6]. The brain, on the other hand, appears to be protected against energy deprivation even when the entire body is under starvation, because nutrients (e.g., amino acids, glucose, and ketone bodies) are compensated by a constant supply from other organs. Therefore, autophagy in the brain neurons does not seem to be induced by energy deprivation, indicating that regulation of autophagy in neurons is different from that in most other cell types [7].

In this study we employed Neuro-2a cell, a neuron-like cell-line, to analyze the regulatory mechanism of autophagy. We found that the function of the Na^+^/H^+^ exchanger (NHE) family of ion transporters affect autophagy in Neuro-2a cells. NHEs are integral membrane proteins catalyzing the exchange of Na^+^ and H^+^ down their respective concentration gradients and play a role in regulating a variety of physiological processes, ranging from the fine control of intracellular pH and cell volume to systemic electrolyte, acid-base and fluid volume homeostasis [8]. Here we showed that expression of NHE1 and NHE5 is correlated to polyQ accumulation levels in cellular and animal models of HD. Together, these data suggest that cellular pH regulation by NHE1 and NHE5 play a role in regulating basal autophagy in the brain's neurons.

## Materials and Methods

### Animals

The technical protocols for animal experiments in this study were approved by Small Animal Welfare Committee at the National Center for Neurology and Psychiatry.

NHE5 null mutant mouse strain was obtained from Deltagen Inc. (Detailed methods and confirmation for the targeted gene deletion are provided by the company upon request.) Mice overexpressing human huntingtin residues 1–171 with 82 glutamine repeats (N171-82Q mice) [9] were obtained from the Jackson Laboratory.

### Cell culture

Neuro-2a cells (ATCC) were maintained using Dulbecco's modified Eagle's medium (Invitrogen) supplemented with 10% fetal bovine serum, 25 U/ml penicillin, 25 µg/ml streptomycin (Invitrogen) and 4 mM L-glutamine (Invitrogen) in a humidifying incubator at 37 C, with 5% CO2 and 95% air, unless otherwise mentioned. Lipofectamine 2000 reagent (Invitrogen) was used for plasmid DNA transfection per manufacturer's protocol. In some experiments, cells were treated with the culture media containing 200 µM DIDS (Sigma) or 10 µM EIPA (Sigma) for 2 hr, or 10 µg/ml E64d (Peptide Institute Inc) and 10 µg/ml pepstatin A (Peptide Institute Inc) for 18 hr prior to morphological or immunoblot analysis.

### Construction of expression plasmids and mutagenesis

pEGFP-C1-MAP1LC3A, pEGFP-C1-MAP1LC3B, pcDNA3-NHE1, and pcDNA3-NHE5 were constructed by amplifying coding regions of the genes by RT-PCR using total RNA extracted from mouse dorsal root ganglion neurons as a template, followed by cloning into the expression plasmids. pcDNA3-NHE1E266I and pcDNA3-NHE5E212I were generated by PCR-based mutagenesis using pcDNA3-NHE1, and pcDNA3-NHE5, respectively, as templates. For construction of dKeima-mem, EGFP region of pEGFP-C1 (Clontech) vector was replaced with dKeima-Red from pdKeima-Red-S1 (Amalgaam) and c-Ha-Ras farnesylation signal (KLNPPDESGPGCMSCKCVLS) was added to the C-terminal using a PCR-based method. NLSQ81EGFP and NESQ81EGFP plasmids were constructed by inserting nuclear localization signal sequence (PKKKRKV)or nuclear exclusion signal sequence (LALKLAGLDI) followed by human atrophin1-derived polyglutamine sequence (81 glutamine with 5 amino acid-flanking sequence derived from atrophin1 at both ends) at the multiple cloning site of pEGFP-N1 plasmid (Clontech) and a myc tag sequence at C-terminus of EGFP by a PCR-based method [10]. Expression plasmids for HttEx1-Q25-EGFP and HttEx1-Q97-EGFP were provided by Dr. A. Iwata (The University of Tokyo), and HttEx1-Q25 and HttEx1-Q97 were constructed by removing EGFP regions from them. The integrity of each clone was confirmed by sequencing.

### Intracellular pH measurements

For intracellular pH estimation using dKeima-mem, fluorescence was measured in standard bath solution containing 140 mM NaCl, 5 mM KCl, 2 mM MgCl_2_, 2 mM CaCl_2_, 10 mM HEPES, and 10 mM glucose at pH 7.4 (adjusted with NaOH). The ratio of florescence intensities of dKeima-Red emissions at 445 nm and 586 nm was calculated for intracellular pH estimation, which was based on previous report on pH measurement using dKeima-red [11]. The high-[K+]/nigericin technique was employed to convert dKeima-Red emission intensity ratios into pH_i_ values [12], [13]. The data shown are obtained from 961 cells in 29 independent experiments for HttEx1-Q25 expressing cells, and from 960 cells in 27 independent experiments for HttEx1-Q97 expressing cells. Differences between groups were examined for statistical significance using Welch's t-test.

**Figure 1:**
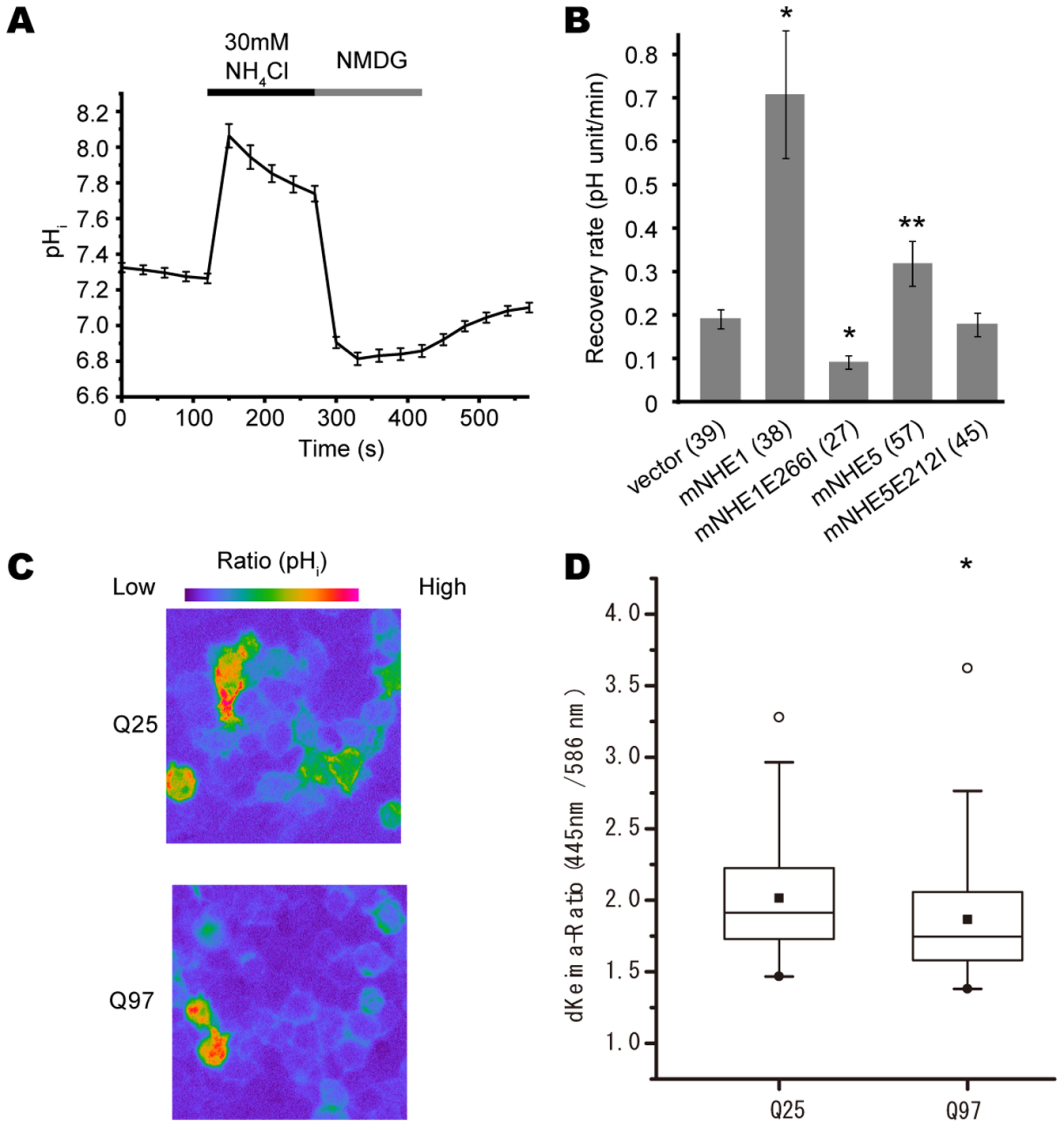
Acidification by polyglutamine protein expression visualized by dKeima-mem-based subcellular pH imaging. A. Representative subcellular pH imaging data in Neuro-2a cells expressing dKeima-mem during acidification followed by recovery in Na^+^-free and Na^+^-containing medium. B. Expression of NHE1 and NHE5 but not by their non-functional mutants facilitates recovery from acidification (see Methods). Mean pH recovery rate ±SD for each type of cells is shown as a bar graph. *P<0.01, **P<0.05 (Student's t-test; in comparison with the recovery rate of vector-only transfected control cells). Numbers in parentheses on X axis indicates the number of cells examined. C, D. Representative intracellular pH imaging results of Neuro-2a cells expressing dKeima-mem together with HttEx1-Q25 or HttEx1-Q97 demonstrated by pseudo-color images (C) and box whisker plot of quantification data 445 nm/586 nm emission ratio(D). In D, boxes represent lower quartile and upper quartile, with bars showing 1.5× of the lower and upper quartile values. White and black circles show max and min, respectively, and quadrangles show median data points. *P<0.001 (Welch's t test).

For characterizing NHEs expressed in Neuro-2a cells, cells were alkalinized and acidified for 2.5 min pre-pulse technique in a solution consisting of 110 mM NaCl, 30 mM NH_4_Cl, 5 mM KCl, 2 mM CaCl_2_, 2 mM MgCl_2_, 10 mM HEPES, 10 mM glucose at pH 7.4 (adjusted with NaOH), followed by incubation for 2.5 min in a Na^+^ free solution containing 140 mM NMDG, 5 mM KCl, 2 mM CaCl_2_, 2 mM MgCl_2_, 10 mM HEPES, 10 mM glucose at pH 7.4 (adjusted with HCl). Recovery of intracellular pH was then observed by another 2.5 min incubation in a Na^+^-containing standard bath solution. Intracellular pH change profile in a representative experiment is shown in Fig 1A. The recovery of pH_i_ was fitted to a single exponential function using Origin Pro 8.0 (OriginLab). “pHi recovery rate” was designated as the rate of Na^+^-dependent intracellular pH (pH unit per min) at 0.05 pH_i_ unit increments from the point of maximum acidification. Differences between groups were examined for statistical significance using Student's t-test.

### Immunofluorescence staining

NHE5 null mutant mice and their wild type littermates (9 weeks of age; 3 males for each genotype) were analyzed. Immunohistochemistry procedures were carried out as described [14]. Mouse anti-HTT a.a. 1–82 monoclonal antibody (Millipore; clone 2B4) and rabbit anti-p62/SQSTM1 polyclonal antibody (MBL) were used as primary antibodies. Alexa 488-conjugated anti-rabbit IgG, andAlexa 594-conjugated anti-mouse IgG (Molecular Probes) were used as secondary antibodies. DAPI (Sigma) was used for identification of nuclei.

### Image analysis for quantification

For analysis using cultured cells, 3 images were obtained from 3 independent experiments for each condition. For aggregate quantification in brain tissues, 6 images of randomly selected fields from cerebellar cortex granular layer sections were obtained from mouse brains of indicated genotypes. Fluorescent images were captured on a laser scanning microscopy (Leica TCS SP2) using ×64 objective lens (each emage is a square, 119.047619 µm each side). Aggregate numbers were counted using PhotoshopCS4 Extended edition. Diameter of aggregate was determined by regarding that the aggregate area is circular. Differences between groups were examined for statistical significance by Student's t-test for aggregate numbers, and by Mann-Whitney's U-test for aggregate diameters.

### RT-PCR analysis

For detection of mRNA expression of NHE family of molecules by RT-PCR in Neuro-2a cells, the following primer sets were used: NHE1-forward (F): CACCAGTGGAACTGGACCTT; NHE1-reverse (R): AAGGTGGTCCAGGAACTGTG; NHE2-F: CAATGACTGCCGTGAAGAGA; NHE2-R: GTCCGAGTCGCTGCTATTTC; NHE3-F CACCACAGGATTGTCCCTCT; NHE3-R: ACAGCAGGAAGGCGAAGATA; NHE4-F: GGCTTTCTCCTGAAGACGTG; NHE4-R: GTCTGTCGCCTTTCCTGAAG; NHE5-F: GCTGAGGGTGAAGAGGAGTG; NHE5-R GGCATAGAGGGCAGAGTGAG.

## Results

### Change in extracellular pH induces autophagosome formation in the Neuro-2a cells stably expressing EGFP-LC3

**Figure 2:**
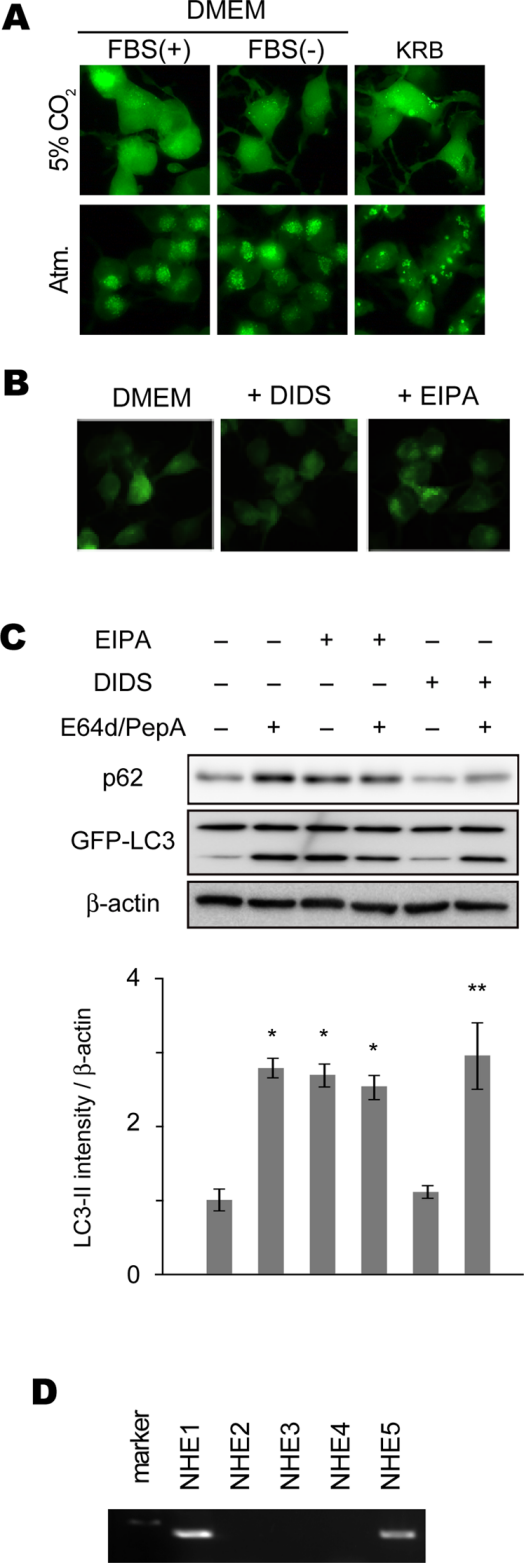
Neuro-2a cells show increased autophagosome formation in response to environmental pH modulation. A, B Representative photomicrographs of Neuro-2a cells stably expressing EGFP-LC3 maintained under indicated medium and atmosphere conditions for 2 hr (A), or with indicated reagents in serum-containing DMEM under 5% CO2 (B). Note the significantly increased autophagosome formation in the cells maintained in Krebs-Ringer bicarbonate buffer (KRB), which contains electrolites and glucose only under 100% normal air, and with EIPA. C Representative images of immunoblot analysis for expression of p62 and LC3 in EGFP-LC3-expressing Neuro-2a cells with indicated reagents. Bar graphs in C show quantification data of LC3-II expression levels relative to the level in EGFP-LC3-expressing Neuro-2a cells with no additional treatment normalized to β-actin expression. Results shown are mean ± SEM. *P<0.01, **P<0.05 (Student's t- test). Note that EIPA-treated cells show increased p62 and LC3-II levels indicating the inhibition of autophagic degradation by NHE inhibition. D. Detection of NHE family members in Neuro-2a cells by RT-PCR.

Previous reports suggested that autophagy in neuronal cells is regulated in a way different from non-neuronal cells [15]. To gain insights on the mechanism of macroautophagy induction in neuron-like cells, we generated mouse neuroblastoma-derived Neuro-2a cells stably expressing EGFP-LC3 and used them as a neuron-like model system. Among physiologically relevant neuronal environmental changes that affect intracellular protein degradation, we chose to examine the effect of environmental pH changes on neuronal autophagy, because lysosomal protein degradation is heavily dependent on cellular pH regulation [16]. To modify environmental pH in cultured cells, we examined Neuro-2a cells stably expressing EGFP-LC3 maintained in an incubator with 100% air (no added CO_2_) for 2 hr. We found that numbers of EGFP-positive spots were significantly increased by incubating the cells under normal air, while EGFP spot formation was not significantly affected by serum deprivation using DMEM lacking serum as culture media (FBS(−)) or by serum/amino acid-deprivation using Krebs-Ringer bicarbonate solution (KRB)(Fig. 2A). This result suggested that pH of culture environment of Neuro-2a cells affects autophagosome formation in them.

To gain insights on the mechanism of this phenomenon, we first tried to analyze which ion channels or transporters are functional in Neuro-2a cells. Major transporters implicated in intracellular pH regulation in various types of cells include sodium-hydrogen exchangers (NHEs/ SLC9 gene family) and several members of bicarbonate transporter superfamily of molecules [17]. Previous reports showed that all the known isoforms of bicarbonate-dependent transporters are inhibited by diisothiocyanostilbene disulfonic acid (DIDS) [18], while members of NHE family are inhibited by 5-(N-ethyl-N-isopropyl)-amiloride (EIPA) [19]. Therefore, we first tried these inhibitors on the Neuro-2a cells. We found that application of EIPA to the Neuro-2a cells in a 5% CO2 incubator significantly increased formation of EGFP-positive spots in the cells, while DIDS showed no effects (Fig. 2B). This result suggested that NHEs play a role in autophagosome formation in the Neuro-2a cells. To distinguish whether EIPA-induced induction of autophagosome formation in Neuro-2a cells is involved in lysosomal acidification only or in induction of autophagy as well, we first performed autophagy flux assays in the presence of EIPA or DIDS (used as a negative control) [20]. We examined expression levels of p62 and LC3-I/II in Neuro-2a cells expressing GFP-LC3 in the presence of EIPA or DIDS, before and after lysosomal inhibitor (E64d/PepA) treatment. We found that EIPA treatment increased both p62 and LC3-II levels, suggesting that EIPA inhibited basal levels of autophagy in Neuro-2a cells, while DIDS did not affect the autophagy level (Fig. 2C). These results suggest that NHEs may affect autophagy levels in Neuro-2a cells. We then examined which family members of NHEs are expressed in Neuro-2a cells by RT-PCR, and found that NHE1 and NHE5 mRNA were detectable, while other members were not (Fig. 2D). Therefore we focused the subsequent analysis on the role of NHE1 and 5.

**Figure 3:**
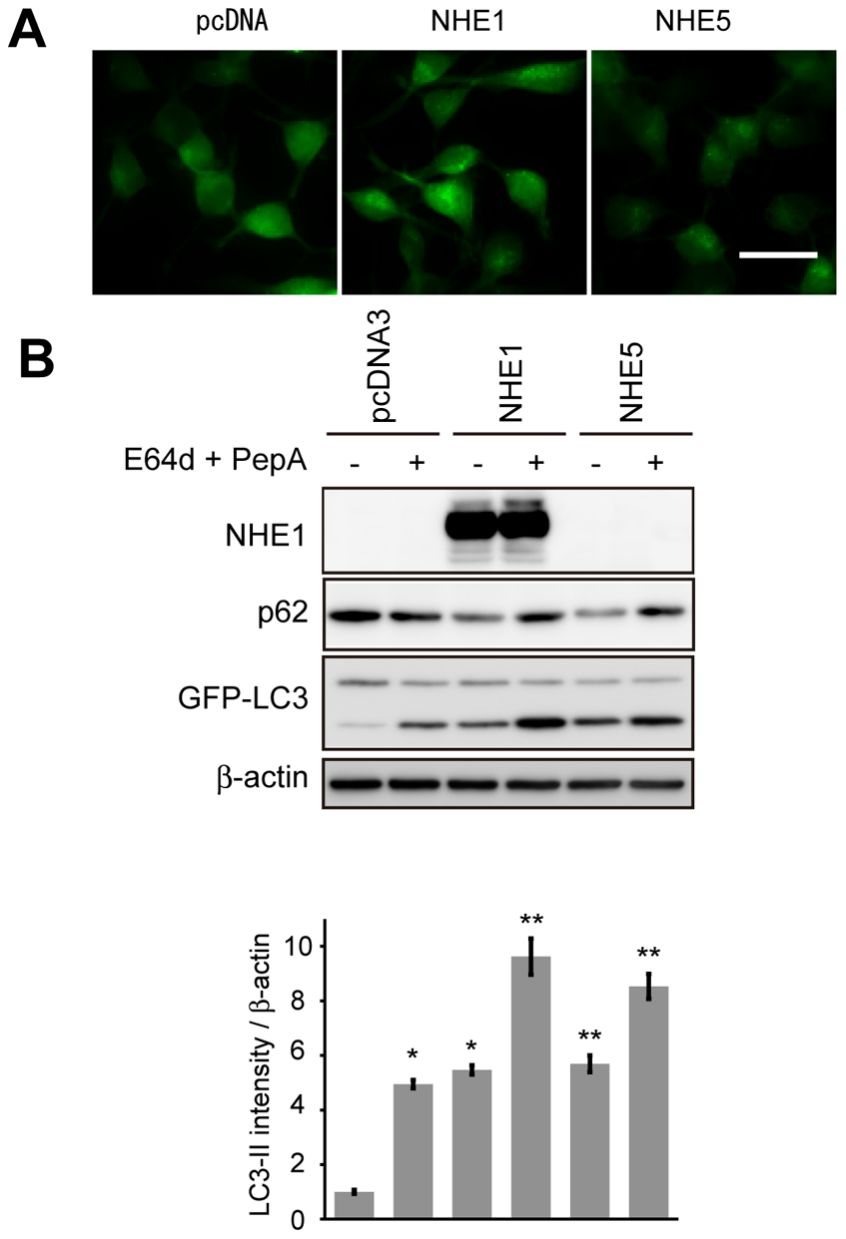
Induction of autophagy in Neuro-2a cells by NHE1 and NHE5. Representative photomicrographs of Neuro-2a cells stably expressing EGFP-LC3 with additional expression of NHE1 or NHE5 (A). Scale bar = 50 µm. Representative images of immunoblot analysis for expression of p62 and LC3 in EGFP-LC3-expressing Neuro-2a cells overexpressing indicated constructs (B). Bar graphs in B show quantification data of LC3-II expression levels relative to the level in EGFP-LC3-expressing Neuro-2a cells with no additional treatment normalized to β-actin expression. Results shown are mean ± SEM. *P<0.01, **P<0.05 (Student's t- test). Note that induced levels of LC3-II by overexpression of NHE1 and NHE5 was further increased by E64d/PepA, suggesting that expression of NHE1 and NHE5 induces autophagy in B.

To further characterize whether pH regulation in Neuro-2a cells by NHE1 and NHE5 is involved in induction of autophagy, we examined whether autophagy is induced in Neuro-2a cells by overexpression of NHE1 or NHE5. For this purpose, we performed autophagy flux assays under overexpression NHE1 or NHE5. To confirm that overexpressed NHE family of molecules can affect intracellular pH as expected, we introduced a pH-sensitive fluorescent dye-based imaging method by using Keima-Red with a plasma membrane localization tag (dKeima-mem). We found that this method is suitable for estimating intracellular pH around physiological level (Fig. 1). By using dKeima-mem, we were able to confirm that overexpression of NHE1 or NHE5, but not NHE1E266I or NHE5E212I, which contains a point mutation to disrupt ion transporting function of each transporter [21], [22], can affect intracellular pH, suggesting that the expression constructs for NHE1 and NHE5 proteins that we used in this study are functional (Fig. 1B). To assess autophagy levels in Neuro-2a cells overexpressing NHE1 and NHE5, we employed Neuro-2a cells stably expressing EGFP-LC3 and examined morphological changes by expression of NHE1 and NHE5. We found that significantly increased number of EGFP puncta, indicative of increased autophagosome formation, by expression of NHE1 and NHE5 (Fig. 3A). We then examined changes in expression levels of LC3-I/II and p62 in Neuro-2a cells by overexpressing NHE1 or NHE5. We found that expression levels of LC3-II are increased in both types of cells (Fig. 3B). To demonstrate that this increase of LC3-II is due to induction of autophagy, we added lysosomal inhibitors (E64d and pepstatin A) to the culture of those cells. We observed an additional increase of LC3-II and p62, suggesting that expression of NHE1 or NHE5 induced autophagy (Fig. 3B). Taken together, these data suggest that NHE family of transporters, NHE1 and NHE5, may function to regulate autophagy levels in Neuro-2a cells by modulating intracellular pH.

### NHE activity prevents polyQ aggregation

Wong et al. reported that expression of HTT proteins with a pathologically extended glutamine repeat in cultured cells causes acidification of culture medium [23]. Together with our current findings, we speculated that expression of abnormal polyQ protein may affect cellular autophagy by modifying intracellular pH, and thereby inhibit cellular protein degradation mechanism to cause abnormal aggregation formation. To explore this possibility, we first examined if expression of abnormally extended polyQ-containing protein changes intracellular pH by employing the dKeima-mem-based pH imaging method (see Methods for detail). We employed htt-Ex1-Q97, containing 97 stretch of glutamine chain in the first exon of HTT, as an abnormal polyQ-containing protein, and htt-Ex1-Q25 as a normal control. We overexpressed them in Neuro-2a cells together with dKeima-mem, and found that Neuro-2a cells expressing htt-Ex1-Q97 showed a relatively low 445 nm/586 nm emission rate (i.e., relatively lower pH) compared with Neuro-2a cells expressing htt-Ex1-Q25 (Fig. 1C,1D), indicating that expression of abnormal polyQ-containing protein acidifies intracellular fluid.

**Figure 4:**
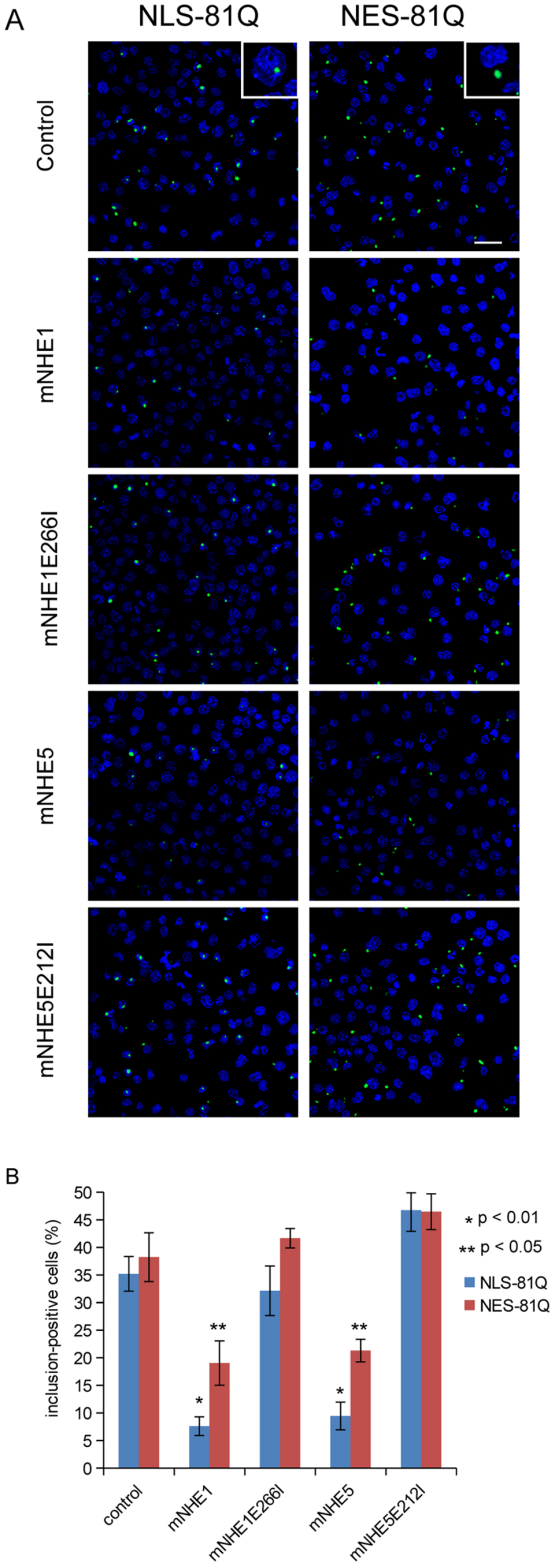
Expression of NHEs inhibits formation of mutant HTT aggregates. Representative photomicrographs of Neuro-2a cells overexpressing plasmids for indicated protein (A), and quantification of the aggregate-positive cell percentage (B). Insets show extranuclear and intranuclear localization of polyQ aggregates by expression of NES-81Q and NLS-81Q, respectively. Scale bar = 30 µm in (A).

To know whether NHE expression affects intracellular polyQ aggregation status, we examined aggregation of polyQ in Neuro-2a cells with or without co-expression of NHE1 or NHE5. PolyQ aggregates are often formed in nuclei, while autophagy-mediated protein degradation occurs in cytoplasm [2]. Therefore, in this experiment, we employed cytoplasmic (NESQ81EGFP-myc) and nuclear polyQ protein (NLSQ81EGFP-myc) expression constructs to examine how expression of NHEs affect the polyQ aggregation derived from each of these constructs. We found that expression of NHE1 or NHE5 suppressed formation of EGFP-labeled polyQ aggregation in cytoplasm and nucleus, while expression of mutant forms of NHE1 and NHE5 did not (Fig. 4). These results suggest that expression of NHE1 and NHE5 function to inhibit polyQ aggregate formation by increasing autophagic degradation of cytoplasmic protein aggregates, which may affect the global polyQ metabolism to decrease nuclear aggregations as well.

**Figure 5:**
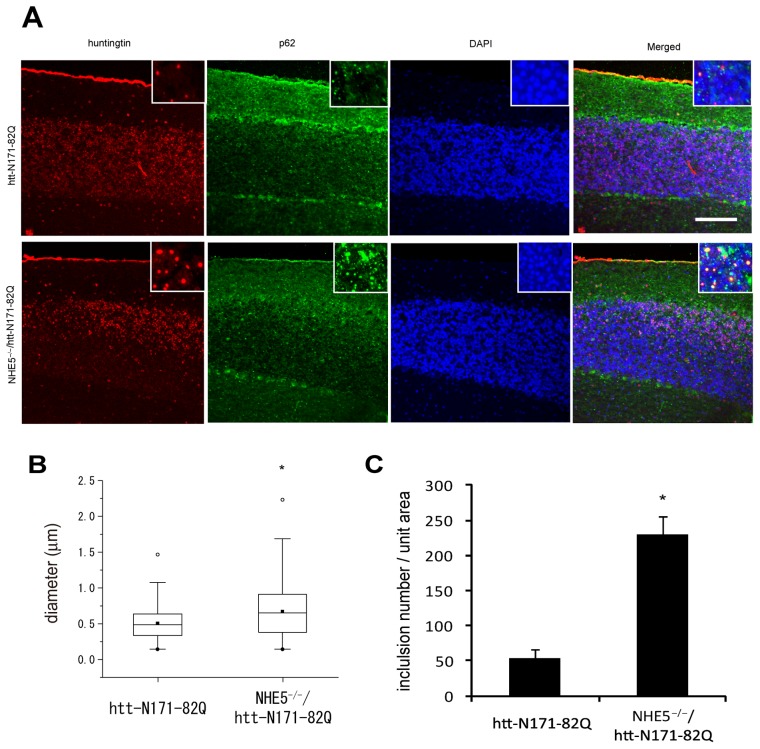
NHE5 null mutation increases polyglutamine protein aggregation in vivo. Representative photomicrographs of polyglutamine protein inclusions formed in cerebellar cortex of mice of the indicated genotype are shown in A. Immunoreactivity of HTT, p62, and DAPI staining images are shown with high-manification view of granular layer inclusions in insets. Scale bar = 30 µm. Quantification of the diameter (B) and number (C) of the aggregates are shown. See Method section for details. In B, boxes represent lower quartile and upper quartile, with bars showing 1.5× of the lower and upper quartile values. White and black circles show max and min, respectively, and quadrangles show median data points. *P<0.001 (Mann-Whitney's U-test). In C, data shown are mean ± SD. *P<0.05(Student's t-test).

To examine the roles of NHEs in more physiological settings, we employed a mouse model polyQ aggregation and examined how it is affected by NHE expression. To this end, we crossed N171-82Q mice (HD model mice overexpressing N-terminal fragment of HTT containing 82 glutamine repeats) with NHE5 knock-out mice. NHE5 knockout mice were generated by insertion of neo-resistance cassette in the exon 6 of mouse NHE5 gene. NHE5 null mutant mice were born with expected Mendelian frequency and did not show gross defect, developmental abnormality, or reproductive failure (data not shown). N171-82Q mice develop nuclear inclusion of mutant HTT in many regions of the brain [9]. To examine the effect of NHE5 deletion in polyQ accumulation in the brain of N171-82Q mice, we examined immunohistochemical distribution of polyQ aggregation in N171-82Q mouse cerebellum with NHE5-null background. From randomly acquired fields, the entire granular layer (from the region right next to the Pukinje cells to medulla) was evaluated for the formation of polyQ aggregation. We found that N171-82Q mouse cerebellar tissues with NHE5-null background showed increased size and numbers of nuclear inclusions (Fig. 5). These data suggest that NHE5 functions to promote degradation of abnormal proteins including polyQ by activating autophagy.

## Discussion

In the current study, we demonstrated that NHEs play a role in maintaining normal neuronal functionality by regulating intracellular pH, and thereby promoting autophagy-mediated degradation of proteins including polyQ-containing molecules. NHE-mediated cellular pH maintenance seemed to contribute to the regulation of basal autophagy levels in neuronal cells. In N171-82Q /NHE5KO mouse cerebellum, we found an increase of polyglutamine aggregation mostly in nuclei of neurons. Previous reports showed that induction of autophagy-mediated protein degradation promotes clearance of cytoplasmic but not nuclear aggregates [3]. However, it is also reported that RNAi-mediated reduction of autophagy machinery may result in a slight increase of nuclear inclusions as well [24]. Our current data suggest that by promoting autophagy-mediated degradation of cytoplasmic aggregation of polyQ, nuclear inclusion can be reduced as well. It is also possible that NHE5 expression may affect other protein degradation system such as the ubiquitin-proteasome system to increase degradation of nuclear inclusions. Further studies will be necessary to explore this possibility.

In this work, we employed NHE5 null-mutant mice to examine *in vivo* role of NHEs in degradation of polyQ, because NHE1 null mutation causes severe developmental defects, including ataxia, growth retardation, and seizures, and results in high mortality rate in early postnatal development [25], [26]. In our experiments using Neuro-2a cell culture, we found both NHE1 and NHE5 show equivalent effect in inducing autophagy, suggesting that NHE1 may also function to promote degradation of polyQ aggregates. To prove this possibility using mouse models, NHE1 conditional KO mice that lack NHE1 in limited regions such as postmitotic neurons may be necessary.

Thus far, it is not clear how the change in cytoplasmic pH induces basal autophagy in neurons. Because intracellular pH is affected by cellular energy production/consumption and metabolic stresses [27], and metabolic changes are key regulators of autophagy [3], it is possible that NHE-induced regulation of autophagy is mediated by a signaling mechanism similar to the regulatory mechanism of energy deprivation-induced autophagy. It is also possible that another novel regulatory mechanism is involved in NHE-induced autophagy, because NHEs affect basal levels of autophagy, which is often regarded as one regulated under a mechanism different from energy deprivation-induced autophagy [3]. Further studies will need to be done to fill the gap between cellular pH regulation and changes in basal autophagy status.
